# Dopaminergic Modulation of Spectral and Spatial Characteristics of Parkinsonian Subthalamic Nucleus Beta Bursts

**DOI:** 10.3389/fnins.2021.724334

**Published:** 2021-11-11

**Authors:** Matthias Sure, Jan Vesper, Alfons Schnitzler, Esther Florin

**Affiliations:** ^1^Institute of Clinical Neuroscience and Medical Psychology, Medical Faculty, Heinrich-Heine University Düsseldorf, Düsseldorf, Germany; ^2^Department of Functional Neurosurgery and Stereotaxy, Medical Faculty, University Hospital Düsseldorf, Düsseldorf, Germany; ^3^Department of Neurology, Center for Movement Disorders and Neuromodulation, Medical Faculty, Heinrich Heine University Düsseldorf, Düsseldorf, Germany

**Keywords:** beta bursts, directional leads, local field potentials, closed-loop DBS, Parkinson’s disease

## Abstract

In Parkinson’s disease (PD), subthalamic nucleus (STN) beta burst activity is pathologically elevated. These bursts are reduced by dopamine and deep brain stimulation (DBS). Therefore, these bursts have been tested as a trigger for closed-loop DBS. To provide better targeted parameters for closed-loop stimulation, we investigate the spatial distribution of beta bursts within the STN and if they are specific to a beta sub-band. Local field potentials (LFP) were acquired in the STN of 27 PD patients while resting. Based on the orientation of segmented DBS electrodes, the LFPs were classified as anterior, postero-medial, and postero-lateral. Each recording lasted 30 min with (ON) and without (OFF) dopamine. Bursts were detected in three frequency bands: ±3 Hz around the individual beta peak frequency, low beta band (lBB), and high beta band (hBB). Medication reduced the duration and the number of bursts per minute but not the amplitude of the beta bursts. The burst amplitude was spatially modulated, while the burst duration and rate were frequency dependent. Furthermore, the hBB burst duration was positively correlated with the akinetic-rigid UPDRS III subscore. Overall, these findings on differential dopaminergic modulation of beta burst parameters suggest that hBB burst duration is a promising target for closed-loop stimulation and that burst parameters could guide DBS programming.

## Introduction

Increased beta band activity in the subthalamic nucleus (STN) is considered to be a hallmark of Parkinson’s disease (PD): It correlates with motor symptoms and is reduced by dopaminergic medication or deep brain stimulation (DBS) in the STN (Ray et al., 2008; Kühn et al., 2009). Recent evidence points to beta activity occurring in phasic bursts in the cortex (Lobb, 2014; Feingold et al., 2015) and within the STN (Tinkhauser et al., 2017a,b). These transient bursts have been suggested to indicate episodes of long-range synchronization in the basal ganglia–cortical circuit (Tinkhauser et al., 2018; Cagnan et al., 2019). Moreover, beta bursts can be used as a feedback signal for closed-loop DBS to improve the stimulation outcome, highlighting their clinical relevance (Arlotti et al., 2018; Velisar et al., 2019). As closed-loop stimulation is still under investigation, different approaches for the feedback signal have been proposed (Swann et al., 2011; Abosch et al., 2012; Lettieri et al., 2012; Little et al., 2013; Priori et al., 2013; Neumann et al., 2014; Qasim et al., 2016; Arlotti et al., 2018; Velisar et al., 2019). In the case of beta bursts, it is not known which burst properties lead to the best clinical outcome if they are used as feedback signal for closed-loop DBS.

Using directional DBS leads as opposed to the spatially unspecific omnidirectional leads we investigate the spatial distribution of STN bursts and analyze whether their characteristics differ within the functional subsystems of the STN. Spatially and functionally, the STN itself can be subdivided into three parts corresponding to the motor, limbic, and associative system (Haynes and Haber, 2013). We also aim for a more precise characterization of STN beta bursts along the frequency dimension. Previously it was demonstrated that PD severity as measured by the motor Unified Parkinson’s Disease Rating Scale (UPDRS III) score, on the one hand, correlates positively with the spectral power in the low beta band (lBB) (Neumann et al., 2016). On the other hand, it correlates positively with the temporal stability of the amplitude in the high beta band (hBB) (Little et al., 2012). At the same time, most PD patients have one spectral power peak in the beta band at an individual frequency (iBP).

Finally, we investigate the effect of dopaminergic medication on STN beta bursts and the relation between burst characteristics and the UPDRS III score. As dopamine alleviates the motor symptoms, a change in burst characteristics due to dopamine would highlight their pathological nature. Such pathological burst parameters would be a good target signal for closed-loop DBS and instrumental to optimize closed-loop STN-DBS.

## Materials and Methods

### Subjects and Surgery

In total 27 (8 female) PD patients (age: 59.0 ± 8.7 years) undergoing surgery for therapeutic STN-DBS in both hemispheres were recruited for this study. Patients had been selected for DBS treatment according to the guidelines of the German Society for Neurology. The Edinburgh Handedness score (81.1 ± 27.0) showed a clear preference for the right side, whereas the side of the main PD impairment was not lateralized (left = 12, right = 12, equal = 3), which was determined by a laterality score based on the UPDRS part III score (Goetz et al., 2008; Heinrichs-Graham et al., 2017). The UPDRS score was assessed 2 days before surgery OFF and ON dopaminergic medication (in the following: OFF and ON).

Written informed consent was obtained from all participants. The study was approved by the local ethics committee (study no. 5608R) and conducted in accordance with the Declaration of Helsinki. DBS electrodes with directional leads were implanted within the dorsal part of each STN at the Department of Functional Neurosurgery and Stereotaxy in Düsseldorf. The implanted DBS electrodes used were the St. Jude Medical Directional lead 6172 (Abbott Laboratories, Lake Bluff, United States) and in one case the Boston Scientific Vercise segmented lead (Boston Scientific Corporation, Marlborough, United States). To enable LFP measurements, the implanted DBS electrodes were externalized using the St. Jude Medical Directional extension 6373 (Abbott Laboratories, Lake Bluff, United States).

The entry point of the STN was identified based on intraoperative microelectrode recordings (Sterio et al., 2002; Moran et al., 2006; Hartmann et al., 2018). Only the height of directional contacts that matched the STN entry point was selected for further analysis. We thus selected only three out of six possible directional contacts, but ensured that the selected contacts were in a comparable anatomic position. Due to a radiopaque marker on the electrode, we identified the segmented contacts facing the anterior, postero-medial, and postero-lateral orientation based on two orthogonal x-ray images. We compared the contacts selected based on the STN entry with the contacts that showed the best clinical outcome. The contact of the best clinical outcome was determined 3–6 months after stimulator implantation and characterized by the best clinical effect due to DBS without any side effects as ascertained by a clinician. In 38% of the cases, the selected contacts were at the height of the clinically chosen contact for therapeutic DBS.

Subthalamic nucleus recordings of four hemispheres were excluded from further analysis because intraoperative microelectrode measurements showed no typical STN activity or the electrode orientation was not visible on the available x-ray images. Additionally, the LFPs of one patient could not be included due to excessive artifacts of unknown origin. In the end, we included LFP recordings from 44 STNs of 24 patients in our analysis.

### Experimental Setup and Recordings

The measurement took place 1–3 (1.3 ± 0.8) days after surgery. The externalized DBS electrodes were connected to an EEG amplifier. All patients were asked to sit relaxed and still. The data were recorded with a sampling rate of 2,400 Hz and a low-pass filter of 800 Hz was applied. The LFP signals were measured against a reference electrode placed at the mastoid. To ensure that patients did not fall asleep, we used an eye tracker, tracking the pupil diameter.

We recorded resting-state activity in three consecutive blocks of 10 min in two conditions for a total of 60 min: once OFF and once ON medication. OFF medication PD oral medication was withdrawn overnight for at least 12 h. In case a patient had an apomorphine pump, this pump was stopped at least 1 h before the measurement. After the three OFF measurement blocks, patients received 1.5 times their levodopa morning dose in the form of rapidly acting dispersible levodopa (173.0 ± 48.9 mg). To ensure a stable ON, we waited for at least 30 min and tested the clinical symptoms before the second half of the measurement. One patient could only be measured ON medication and one only OFF.

### Signal Processing

All data processing and analyses were performed using MATLAB (version R 2016b; MathWorks, Natick, United States). Custom-written MATLAB scripts and the toolbox Brainstorm^1^ (Tadel et al., 2011) were used. To ensure artifact-free data, two persons independently inspected the data visually, cleaned artifacts, and compared the cleaned output. In case of differences, the questioned time segment was rejected. The line noise was removed from all channels with a notch filter with a 3-dB bandwidth of 1 Hz at 50, 100, 150, …, 550, and 600 Hz. The LFP recordings from the DBS electrode were re-referenced against the mean of all LFP channels. Noisy or flat LFP channels were excluded from further analysis. Time segments containing artifacts were removed from the time series, but if artifacts just occurred frequently in one channel, only this whole channel was removed. All data were high-pass filtered with 1 Hz to remove movement-related artifacts. Furthermore, the data were down-sampled to 1,000 Hz. To avoid the influence of different impedance values between patients and recording sessions, we finally calculated the z-transformation of the preprocessed time series separately for each recording session.

### Detection of Bursts in the Beta-Frequency Range

Within the beta band, different activity patterns have been described for the lower and higher beta-frequency range (Priori et al., 2004; Kühn et al., 2006). As the definition and segmentation of the beta band differ among research groups, we decided to divide the beta band into a lower and a higher sub-band of equal size (12–24 Hz and 24–35 Hz). Moreover, we considered a ±3-Hz band around the iBP (mean ± SD: 22.1 ± 5.8 Hz) of each patient. The iBP was determined OFF medication based on the beta peak in the individual power spectrum. For this purpose, the power spectrum in the beta band was examined for local maxima. In case the maximum was at the corner frequencies of 12 Hz or 35 Hz, the amplitude at 11 or 36 Hz needed to be lower for the iBP to be considered at 12/35 Hz. The maximum with the highest amplitude in all contacts of one patient was considered as iBP frequency. ON medication, the beta peak was generally reduced or vanished completely for some patients. In case a peak was still visible ON medication, it was always within 1 Hz of the OFF peak, i.e., covered by our ±3-Hz interval. The power spectra were determined based on the *z*-score normalized time series with 1-Hz resolution using the Welch method with a window length of 1 s and an overlap of 50% (Welch, 1967). To compare different spectra, we corrected for the 1/f characteristic of the LFP signal and normalized to the total power of 5–45 Hz and 55–95 Hz analog to Neumann et al. (2016).

The preprocessed LFP data were used to detect bursts within the two beta sub-bands and the iBP. Our approach follows Tinkhauser et al. (2017a, 2017b), but we determined the bursts based on the *z*-value normalized data rather than the raw data. Afterward, following the burst detection approach by Tinkhauser et al. (2017a, 2017b), Morlet wavelets (Tallon-Baudry et al., 1997) as implemented in Brainstorm were calculated for the lBB, the hBB, and around the iBP. The time-evolving amplitude was smoothed by a 200-ms moving average, followed by a DC-offset correction with a time constant of 20 s to correct for a potential baseline offset. For each patient, channel, and frequency, we calculated the 75th percentile of the OFF and ON time series and took the average of both of them. The separate *z*-score normalization of the LFP data OFF and ON medication could potentially mask the differences in the burst amplitudes between OFF and ON. Despite the *z*-value normalization, there were significant differences in beta power. Moreover, the bursts were detected based on a common threshold from the combined ON and OFF recording, which ensures that differences in burst amplitude between OFF and ON can be detected.

For a time point to be part of a burst in the respective frequency band, the amplitude needed to be higher than the 75th percentile. All consecutive time points with an amplitude exceeding the threshold were assigned to the same burst. The minimal burst duration was set to 80 ms, which is equivalent to two oscillatory cycles at 24 Hz. For every burst, the time point of the amplitude crossing the threshold and again dropping below were stored. The value of the maximum burst amplitude and its time of occurrence were also stored. Due to the applied burst detection scheme, we are referring to the power based on the *z*-score transformed time series and not to the power of the raw time series when we are considering the burst amplitude.

### Statistical Analysis

For the number of bursts per minute which is calculated by the total number of bursts detected for one channel divided by the total recording time in minutes (in the following: burst rate), burst duration, and amplitude, we compared the recording orientation of the LFP contacts, frequency band, and the medication state. Therefore, we performed a three-way ANOVA (Yates, 1934) in MATLAB. The dependent variables were burst rate, duration, and amplitude, respectively, and the independent variables were directions (anterior, postero-medial, and postero-lateral), frequency bands (iBP, lBB, and hBB), and medication states (OFF and ON). Because the correlation of the burst parameters between the hemispheres was partially significant, but a paired *t*-test showed no significant differences between hemispheres, the evidence on hemisphere dependence is inconclusive. Following the previous literature (Zavala et al., 2017), we opted to pool both hemispheres. Therefore, the incoming sample size for ANOVA was the total number of good LFP data by orientation (anterior: OFF and ON each of the 31 LFPs; postero-medial: OFF and ON each of the 37 LFPs; postero-lateral: OFF 31 LFPs and ON 32 LFPs). For the *post hoc* test, a *t*-test was used, which was corrected for multiple comparisons using the Bonferroni method, again using the MATLAB implementation. We corrected for two medication states, three frequency bands, and three contact directions for a total of 18 comparisons.

Finally, the Pearson correlation between the akinetic/rigid (AR) UPDRS III subscore (sum of the 13 items 3.3 a–c, 3.4 ab, 3.5 ab, 3.6 ab, 3.7 ab, and 3.8 ab) and the beta burst characteristics, as well as the power values from the power spectra, were calculated OFF medication. All reported correlation *p*-values are Bonferroni corrected for the three contact orientations and the three frequency bands.

## Results

### Beta Power

**FIGURE 1:**
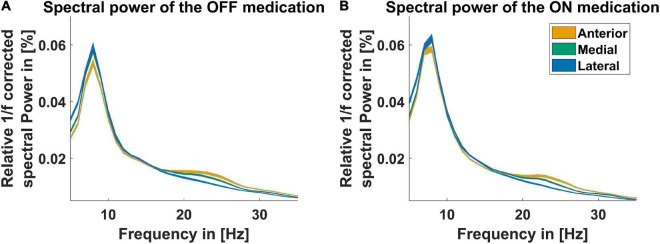
Power spectra for three recording directions. The spectra displayed for the contact orientation anterior (orange line), postero-medial (green line), and postero-lateral (blue line) are corrected for the 1/f characteristic of the LFP signal and normalized to the total power from 5–45 Hz to 55–95 Hz. The *x*-axis presents the frequency from 5 to 35 Hz. **(A)** Shows the spectra OFF medication and **(B)** shows the spectra ON medication. The colored shaded areas indicate one standard deviation of the mean.

Figure 1 displays the average power spectra OFF and ON medication across patients for the three different LFPs at the STN entry from 5 to 35 Hz. The power spectra are 1/f corrected and normalized to the total power of 5–45 Hz and 55–95 Hz. There were no significant differences between power of the different recording orientations in each medication condition but between the power OFF and ON medication. The difference was significant in the anterior direction from 32 to 34 Hz, the postero-medial one from 24 to 28 Hz, and the postero-lateral one from 31 to 34 Hz. Beta peaks OFF medication occurred mainly around 24 Hz at the anterior and postero-medial contact (Figure 1). As the beta peak frequency differed between the recording directions, we investigate in the following to what extent the recording orientation and frequency band influence beta bursts.

### Burst Characteristics

**FIGURE 2:**
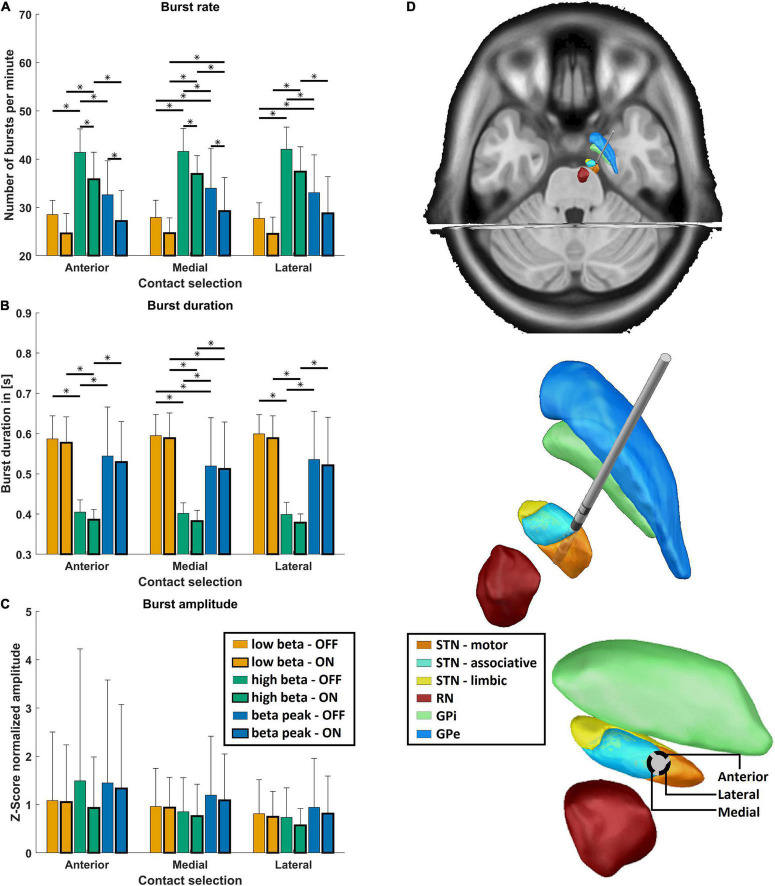
Burst characteristics. **(A)** Shows the mean burst rate, **(B)** shows the mean burst duration, **(C)** shows the mean burst amplitude, and **(D)** shows the exemplary reconstructed electrode position of the right hemisphere. **(A–C)** The mean values for the three frequency bands (low beta band in orange, high beta band in green, and the individual beta peak frequency in blue) are shown OFF (thin frame) and ON medication (bold frame) grouped on the *x*-axis for the anterior, postero-medial, and postero-lateral directions for each burst parameter. Error bars depict one standard deviation. Stars indicate significant differences with *p* < 0.05 after Bonferroni-corrected *post hoc* tests between the respective groups. **(D)** Shows the reconstructed electrode on top, with focus on the surrounding subcortical structures (STN, subthalamic nucleus divided into motor, associative, and limbic areas; RN, red nucleus; GPi, globus pallidus pars internus; GPe, globus pallidus pars externus). At the bottom, the perspective is along the electrode so that the directions of the electrode contacts can be seen.

Figure 2 illustrates characteristics of STN beta bursts depending on medication state, pre-defined frequency band, and electrode contact orientation. Panel A displays the burst rate for the two medication states, the three contact orientations, and frequency bands. Medication had a significant main effect indicating that dopaminergic medication decreased the burst rate for all orientations and frequency bands [*F*(1,579) = 97.1, *p* = 2.8E-21, η^2^p = 0.144]. This was also evident in the *post hoc* test for the hBB and iBP in anterior and postero-medial orientation (*p* < 0.05). Thus, a high burst rate seems to be a characteristic of PD pathology. In addition, there was a significant main effect of frequency for the burst rate [*F*(2,579) = 283.9, *p* = 1.2E-86, η^2^p = 0.495] but no significant main effect of the contact orientation. Based on *post hoc* tests, the hBB burst rate was higher compared to the lBB and the iBP for all contact orientations and both medication states (*p* < 1.0E-6; Figures 2A,B). The rate at the iBP was only significantly higher compared to the lBB OFF medication for the postero-medial and postero-lateral direction and ON medication only for the postero-medial direction (*p* < 0.05; Figure 2A).

Medication had also a significant main effect for burst duration, which was reduced due to dopaminergic medication [*F*(1,579) = 4.6, *p* = 0.03, η^2^p = 0.144], which can be seen in Figure 2B. Moreover, the burst duration was influenced by the frequency band but not by the contact orientation [*F*(2,579) = 341.6, *p* = 1.0E-98, η^2^p = 0.495]. *Post hoc* analysis revealed that the burst duration was significantly shorter for the hBB compared to the lBB as well as the iBP in both medication states (*p* < 1.0E-7; Figures 2C,D). The burst duration was also significantly shorter at the iBP compared to the lBB (*p* < 0.01; Figures 2C,D) but only for the postero-medial direction both OFF and ON medication. Interestingly for the burst amplitude, the contact orientation had a significant main effect across medication states and frequency bands [*F*(2,579) = 6.7, *p* = 0.001, η^2^p = 0.003] but not for the medication and frequency itself.

### Clinical Relevance of Burst Characteristics

**FIGURE 3:**
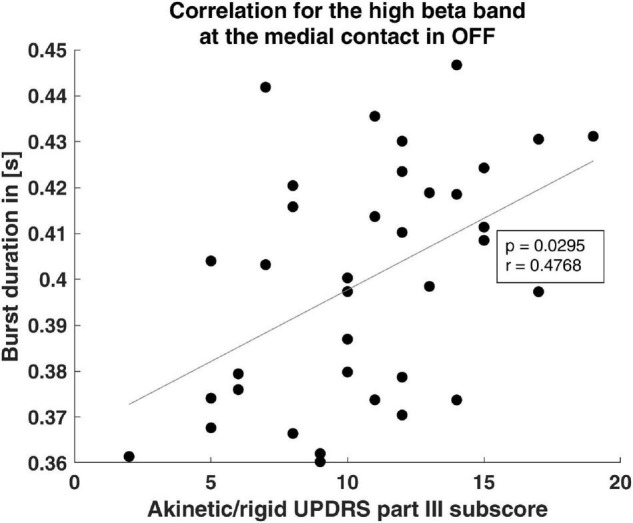
Clinical correlation of burst duration. The scatter plot shows the significant correlation of the burst duration and the akinetic/rigid UPDRS part III subscore. The gray line indicates the linear fit.

Because a positive correlation between bradykinesia and overall beta oscillations has previously been reported (Ray et al., 2008), we tested whether beta burst characteristics correlate with the AR UPDRS subscore. We focused on the AR UPDRS subscore, because, based on the UPDRS score, 70.4% of our patients were of the akinetic-rigid subtype. The OFF hBB burst duration of the postero-medial contact was significantly positively correlated with the OFF AR subscore (*p* = 0.03, *r* = 0.48; Figure 3). For the other contact orientations and frequency bands, there was no significant correlation.

## Discussion

To our knowledge, this is the first work to investigate the spatial characteristics of STN beta bursts recorded with directional electrodes. Spatially, we found a non-homogeneous distribution of the burst amplitude. However, we could not identify any outstanding direction with respect to the amplitude or the rate and duration of the bursts. However, the burst rate and duration in particular are frequency-dependent, which makes frequency selection very important for applications such as closed-loop DBS.

### Slight Directionality of the Beta Burst Amplitude

It has been shown that for each patient, a preferential contact exists in terms of best clinical outcome and many patients benefit from directional stimulation (Gordon et al., 2017; Hartmann et al., 2019). Therefore, we wanted to identify whether beta burst properties differ between the three recording orientations and thus could serve as an indicator for stimulation selection. There was a main effect of recording orientation for burst amplitude. However, *post hoc* tests did not reveal one prominent direction for amplitude or any other measure. Therefore, based on our analysis, no particular anatomic direction can be recommended for directional DBS. The reason for this could be that all contacts used were placed in the dorsal end of the STN, which is functionally attributed to the motor system (Parent and Hazrati, 1995). Therefore, a comparable neurophysiological signal at all contact orientations is plausible and suggests a homogeneous structure of this area. In line with this reasoning, the connectivity between STN and cortex is functionally organized based on the functional subareas of the STN (Lambert et al., 2012). Nevertheless, there is a significant main effect of direction for the burst amplitude. One possibility is that the anatomical dependence of the bursts will only reveal itself with a larger number of cases.

### Beta Burst Activity Is Frequency Specific

We considered three different frequency bands within the beta band: the lBB, the hBB, and the frequency range around the individual beta peak frequency. The reason for this choice was that previous publications reported results specifically for sub-bands of the beta band (Priori et al., 2004; Little et al., 2012; Tinkhauser et al., 2017a,b). We found that burst characteristics differ depending on the chosen frequency band. The burst rate increases for higher frequencies, while the duration decreases. In line with this finding, the AR score correlated with the burst duration in the hBB but no other frequency band. In addition, duration was significantly reduced by dopaminergic medication only for the hBB and iBP. This suggests that bursts in the hBB are likely linked to the pathophysiology of PD, which is consistent with the results of Little et al. (2012). However, it contrasts with the results of Priori et al. (2004) and Neumann et al. (2016), but these studies considered beta power, not beta bursts. A pairwise test of power values compared to Priori et al. (2004) showed significant influence of dopaminergic medication on power in the hBB but not in the lBB.

To further investigate the importance of the frequency band definition, we performed our analysis with a modified frequency band definition, assigning the lBB to 12–20 Hz and the hBB to 21–35 Hz. When comparing the results for the two frequency band definitions, there were no differences with respect to significant findings for the medication dependence or the direction dependence of the burst parameters. However, while for the frequency band separation at 24 Hz there were no significant differences for the burst amplitude between the frequency bands, for the separation at 20 Hz, there were significant differences in the burst amplitude between the lBB and iBP and between hBB and iBP for both medication states and all three directions. In addition, significant differences for burst rate and duration were found between the lBB and iBP. This change in findings based on the beta band separation is most likely due to beta peaks formerly assigned to the lBB now being assigned to the hBB with the separation at 20 Hz. This difference in results suggests that if a beta peak occurs in the power spectra, the frequency band around this peak should be favored for closed-loop stimulation for example; if no peak is present, our results indicate that the hBB could be a good alternative.

### Burst Duration Best Suited as Stimulation Trigger in Closed-Loop Deep Brain Stimulation

Burst characteristics OFF medication are linked to the pathology of PD, while those ON medication approximate physiological activity. As expected based on previous publications, we could find an effect of dopaminergic medication on beta bursts (Ray et al., 2008; Kühn et al., 2009; Tinkhauser et al., 2017a,b). Since we could detect a main effect only with respect to the duration and the rate of the bursts, this suggests that these are more pathologically altered by PD than the amplitude of the individual bursts. The burst amplitude is a necessary quantity for the burst detection method employed in the present study. However, according to our results it is less suitable for distinguishing between pathological and physiological bursts.

In contrast, it has previously been described that a long burst duration is being positively correlated and a short burst duration is being negatively correlated with clinical motor impairment (Tinkhauser et al., 2017a). In our study, only the duration significantly correlated with the AR score and the duration was reduced under medication. Therefore, burst duration seems to be more tightly linked to PD motor symptoms than the burst amplitude and rate. This suggests that the burst duration is the best candidate for a stimulation trigger in closed-loop DBS. This conjecture is in line with previous results that reduced burst duration is associated with improved movement velocity due to DBS (Kehnemouyi et al., 2020). However, since amplitude is the key parameter in burst detection, further studies are needed to understand the interplay of amplitude and burst duration for detecting pathological bursts and their usefulness as stimulation triggers in closed-loop DBS.

To use electrophysiological signals as control parameters for closed-loop DBS, they should remain stable over months and years. We recorded the LFP signals a few days after electrode implantation, when the tissue is still subject to transient processes such as inflammation, which may affect recording properties and neuronal activity. However, previous studies indicate that STN beta band LFP patterns and response profiles stay almost unchanged for years after DBS electrode implantation (Abosch et al., 2012; Giannicola et al., 2012). Moreover, beta activity continues to correlate with severity of PD motor symptoms 8 months after implantation (Neumann et al., 2017). Therefore, it is likely that the duration of beta bursts remains stable over a long period of time and thus provides a valid control parameter for closed-loop DBS. Still, the stability of beta burst duration over longer time periods needs to be investigated in future studies.

### Limitations

Because we at the latest recorded the LFP data 3 days after the DBS surgery, our recordings might be affected by the stun effect (Chen et al., 2006). Due to the magnitude of the stun effect being unknown and immeasurable, it is impossible to correct the electrophysiological data for it. In line with other studies, the UPDRS values were collected before the DBS surgery and thus do not capture the stun effect on the clinical symptoms. This timing difference likely also influences the calculated correlation between the UPDRS and the beta burst parameters. A further limitation is our assumption that beta bursts in one STN arise independently from the other STN. Based on our data, the evidence on the STN activity being independent of the hemisphere was inconclusive. Our decision to pool the data follows previous literature treating the LFPs of both STNs as independent (e.g., Zavala et al., 2017).

### Conclusion

Using directional contacts, we intended to identify the spatial distribution of beta bursts at the entry point of the STN. However, based on the electrode’s recording orientation, we could not identify one orientation with significantly different burst parameters than the other orientations, even though there was an overall effect of orientation for the burst amplitude.

Still, we could identify a strong frequency dependence of beta bursts. Correlation with the akinesia and rigidity scores indicates in particular that hBB burst duration is pathologically increased. In addition, dopaminergic medication influences burst rate and duration. These two findings speak in favor of the hBB bursts as feedback signal for stimulation in closed-loop DBS.

## Data Availability Statement

The raw data supporting the conclusions of this article will be made available by the authors, without undue reservation.

## Ethics Statement

The studies involving human participants were reviewed and approved by the Ethics Committee, Medical Faculty, Heinrich Heine University Düsseldorf. The patients/participants provided their written informed consent to participate in this study.

## Author Contributions

MS: software, validation, formal analysis, investigation, data curation, writing—original draft, and visualization. JV: resources and writing—review and editing. AS: methodology, resources, and writing—review and editing. EF: conceptualization, methodology, validation, formal analysis, investigation, data curation, writing—review and editing, supervision, project administration, and funding acquisition. All authors contributed to the article and approved the submitted version.

## Conflict of Interest

AS has been serving as a consultant for Medtronic Inc., Boston Scientific, St. Jude Medical, and Grünenthal, and received lecture fees from AbbVie, Boston Scientific, St. Jude Medical, Medtronic Inc., and UCB. The remaining authors declare that the research was conducted in the absence of any commercial or financial relationships that could be construed as a potential conflict of interest.

## Publisher’s Note

All claims expressed in this article are solely those of the authors and do not necessarily represent those of their affiliated organizations, or those of the publisher, the editors and the reviewers. Any product that may be evaluated in this article, or claim that may be made by its manufacturer, is not guaranteed or endorsed by the publisher.

## Footnotes

1
http://neuroimage.usc.edu/brainstorm/Introduction

## Abbreviations

STN: subthalamic nucleus
PD: Parkinson’s disease
DBS: deep brain stimulation
UPDRS III: motor Unified Parkinson’s Disease Rating Scale
lBB: low beta band
hBB: high beta band
iBP: individual beta peak frequency.
